# Efficiency of Wheat Straw Biochar in Combination with Compost and Biogas Slurry for Enhancing Nutritional Status and Productivity of Soil and Plant

**DOI:** 10.3390/plants9111516

**Published:** 2020-11-08

**Authors:** Aown Abbas, Muhammad Naveed, Muhammad Azeem, Muhammad Yaseen, Rehmat Ullah, Saud Alamri, Qurrat ul Ain Farooq, Manzer H. Siddiqui

**Affiliations:** 1 Institute of Soil and Environmental Sciences, University of Agriculture Faisalabad, Faisalabad 38040, Punjab, Pakistan; aownabbas15@yahoo.com (A.A.); dr.yaseen@gmail.com (M.Y.); 2 College of Natural Resources and Environment, Northwest A & F University, Yangling 712100, China; azeem@nwafu.edu.cn; 3 Department of Agriculture, Soil and Water Testing Laboratory, Dera Ghazi Khan 32200, Pakistan; rehmat1169@gmail.com; 4 Department of Botany and Microbiology, College of Science, King Saud University, Riyadh 11451, Saudi Arabia; saualamri@ksu.edu.sa; 5 Phytophthora Science and Management, Environmental and Conservation Sciences, Murdoch University, Murdoch, WA 6150, Australia; anniepk_elegant@yahoo.com; 6 Institute of Agricultural Sciences, University of the Punjab, Lahore 54590, Punjab, Pakistan

**Keywords:** wheat straw biochar, biogas slurry, compost, soil health, maize

## Abstract

In the present study, we investigated the impact of different combinations of wheat straw biochar, compost and biogas slurry on maize growth, physiology, and nutritional status in less productive soils. The experiment was performed as a completely randomized block design in a greenhouse pot experiment. The compost and biogas slurry were applied with and without biochar. The results revealed that a combination of biochar, compost, and biogas slurry enhanced the cation exchange capacity (31%), carbon (83%), phosphorus (67%) and potassium (81%) contents in the soil. Likewise, a significant increase in soil microbial biomass carbon (15%) and nitrogen (37%) was noticed with the combined use of all organic amendments. Moreover, the combined application of biochar, compost and biogas slurry enhanced soil urease and β-glucosidase activity up to 96% and 67% over control respectively. In addition, plant height, chlorophyll content, water use efficiency and 1000-grain weight were also enhanced up to 54%, 90%, 53% and 21% respectively, with the combined use of all amendments. Here, biochar addition helped to reduce the nutrient losses of compost and biogas slurry as well. It is concluded that biochar application in combination with compost and biogas slurry could be a more sustainable, environment-friendly and cost-effective approach, particularly for less fertile soils.

## 1. Introduction

Nutrient depletion from soils accelerated by nutrient mining, imbalanced use of fertilizers and poor land management practices pose serious threats to food security [1,2]. Poor nutrient use efficiency (NUE) of crops causes a significant decline in soil quality and crop yields in developing countries [3,4]. Inorganic fertilizers significantly boost crop yield, and their application has steadily increased due to per capita land shrinking and deterioration in soil quality [5]. Inorganic fertilizers alone cannot sustain crop production and their excessive use has caused environmental problems. Eutrophication has been caused by nutrients leaching into the water bodies [6,7]. Similarly, extensive farming practices have reduced soil organic matter (SOM) contents thereby increasing cation exchange capacity (CEC) of soils and lowering fertilizer use efficiency [8]. Maintaining an optimum level of nutrients through management practices and organic amendments play a significant role in sustainable crop production and improving soil fertility [9]. The addition of organic amendments in soils offers economic and environmentally friendly approaches for sustainable crop production [10]. Organic matter in the soil has a pivotal role in biological nutrient cycling and soil management. The decline in SOM due to high temperature and nutrient mining leads to soil degradation processes, such as nutrient deficiency, erosion, salinization, compaction, destruction of microbial population, and even desertification [11]. Low organic matter soils have lost soil carbon which causes wastage of agriculture inputs and loss of crop productivity [12].

Various organic amendments such as cover crops, animal manure, mulches, or compost have been used to enhance nutrients cycling, microbial activities, the utilization efficiency of mineral fertilizers, and to reduce soil erosion, and suppress diseases and other soil born pathogen [10]. It was observed that soil amended with manure and compost has a higher content of organic carbon (OC), macronutrients (P, K, Ca, Mg), and soil microbes, as compared to un-amended soil [13]. It is well documented that compost addition in soil improves soil structure resulting in reduction in bulk density, and an increase in soil aeration and water retention [14]. However, toxic constituents such as heavy metals [15] and other organic compounds in compost may contaminate surface water bodies through runoff and percolation of contaminated water. It limits the utilization of compost [16]. Adding to these, biogas slurry (BS) is a secondary product produced after the digestion of animal dung in the process of biogas production [17]. It is a rich source of nutrients (micro and macronutrients), amino acids, humic acid, hormones, vitamins, and other low-molecular-mass bioactive substances [18]. It was observed by many researchers that biogas slurry addition in soil enhanced nitrogen (N) uptake [19,20], improved microbial diversity, soil microbial biomass (SMB) [21], and increased crop production [22,23]. However, some limitations are associated with organic amendments application in the soil such as low yield, as compared to chemical fertilizers [24]. These amendments provide short term benefits to crop and get decomposed in one cropping season due to high temperature [25]. 

Conversion of easily decomposed organic matter into biochar is an alternative approach to tackle these challenges [26,27]. Biochar addition not only improves nutrient use efficiency (NUE) and fertilizer use efficiency (FUE), but also resists soil carbon (C) decomposition [28]. Biochar is a carbonaceous dark-colored porous material produced during the thermal degradation of organic compounds in the absence of oxygen [29]. A variety of feedstock such as agricultural wastes (straw, rice hulls, nutshells, tree bark, wood chips, and switchgrass), animal wastes and bones etc. are used in biochar production [29,30]. Physiochemical properties of biochar have an advantage over other organic materials. These promote long-term carbon persistence in the environment and enhanced soil nutrient status [31]. Due to large surface area, it plays a significant role in improving soil properties like an increase in NUE, mitigation of biotic and abiotic stresses, improvement in soil quality, and increase in crop yield [32]. Biochar addition in soil enhances OC, water holding capacity (WHC) [33] soil aeration, microbial activity and biodiversity [29], nutrient holding capacity and availability, and reduce nutrient leaching [28]. It results in improvement of fertilizer use efficiency, C sequestration [34] and a decrease in metal toxicity in contaminated soils [30]. 

The sole application of biochar, compost, and biogas slurry has been tested on crop growth and soil qualities by many researchers [35,36,37]. However, few studies have been reported to determine the effects of combined use of biochar, compost, and biogas slurry on the growth and yield of maize under nutrient-depleted soils. We hypothesize that biochar application will help to reduce the nutrient losses of compost and biogas slurry by providing large surface area, enhancing cation exchange capacity, and reducing the toxic effects of compost. Additionally, biochar will provide a suitable habitat for soil microbes by supplying essential nutrients for a longer period not only for microbes but also for the plant which may consequently enhance plant growth. The present trial was conducted to assess the effect of biochar, compost, and biogas slurry with different combinations on the nutritional status of soil, and growth and yield of a maize plant.

## 2. Results

### 2.1. Soil Properties Influenced with Organic Amendments

The results of soil chemical properties after harvesting of maize revealed that organic amendments significantly enhanced the chemical properties such as electrical conductivity (EC), cation exchange capacity (CEC), soil organic carbon (SOC), available phosphorus (P), and potassium (K) of soil, while pH was found to be reduced with the biochar addition (Table 1). Soil without biochar amendment increased soil EC (13%), CEC (19%), SOC (67%), P (58%), and K (34%) in T4 treatment (biogas slurry 0.5% (*wt/wt)* in combination with compost 0.5% (*wt/wt*) (BS_0.5_ + CM_0.5_)) with respect to control. However, in biochar amended soil the highest increase was observed in EC (14%), CEC (31%), SOC (83%), P (67%), and K (81%) with T8 treatment comprising of consortium of biochar, biogas slurry, and compost (WSB_0.3_ + BS_0.3_ + CM_0.3_), as compared to control. While a significant reduction in pH (0.15 unit) was noticed with T8 treatment, as compared to T1 treatment set as control.

### 2.2. Nutritional Status of Maize

The effects of biogas slurry and compost (with and without biochar) addition on the nutritional status of maize are presented in Table 2. A significant increase in the nutrient contents (N, P, and K) of shoot and grain was observed with biochar alone (T5) and in combination with compost and biogas slurry (T8). Without biochar treatments, the highest increase in shoot N (72%), P (43%), K (22%), and grain N (63%), P (84%), and K (65%) was observed in T4 treatment (BS_0.5_ + CM_0.5_) over control. However, with biochar application, the highest increase in shoot N (96%), P (57%), K (49%), and grain N (85%), P (97%), and K (92%) was noticed in T8 treatment representing combined application of biochar, compost, and biogas slurry(WSB_0.3_ + BS_0.3_ + CM_0.3_), as compared to control treatment (T1).

### 2.3. Physiological Parameters 

The separate and cumulative effects of biogas slurry and compost in combination with biochar on maize physiology are given in Table 3. The results revealed that organic amendments significantly decreased the physiological parameters such as relative water content (RWC), electrolyte leakage (EL), transpiration rate (TR), chlorophyll contents (CC), stomatal conductance (SC) and water use efficiency (WUE). Without biochar treatments, the highest decrease in RWC (15%), EL (23%), TR (44%), CC (70%), SC (39%), and WUE (76%) was noticed in T4 treatment (BS_0.5_ + CM_0.5_), when compared to control. However, with biochar application, the highest decrease in RWC (23%), EL (39%), TR (77%), CC (90%), SC (49%), and WUE (97%) was noticed in T8 treatment, representing consortium of biochar, biogas slurry, and compost (WSB_0.3_ + BS_0.3_ + CM_0.3_), as compared to control treatment (T1).

### 2.4. Maize Growth Parameters

The effects of biochar application along with biogas slurry and compost amendments on maize growth parameters are given in Table 4. In the present study, organic amendments significantly enhanced the SL, SFW, SDW, LA, CL, GY, and 1000-GW of maize plant. The highest increase in SL (56%), SFW (68%), SDW (70%), LA (46%), CL (30%), GY (47%), and 1000-GW (38%) was noticed in T4 treatment without biochar (BS_0.5_ + CM_0.5_). However, with biochar application, the highest increase in SL (68%), SFW (91%), SDW (92%), LA (64%), CL (76%), GY (51%), and 1000-GW (43%) was noticed in T8 treatment comprising of consortium of biochar, biogas slurry, and compost (WSB_0.3_ + BS_0.3_ + CM_0.3_), as compared to control (T1).

### 2.5. Effects of Organic Amendments on SMB and Enzyme Activity 

The effects of biogas slurry and compost (with and without biochar) addition to soil on microbial biomass and enzyme activity are presented in Table 5. The results revealed that organic amendments significantly enhanced the SMBC, SMBN, UA, BGA, and APA as evidenced from Table 5. The highest increase in SMBC (7%), SMBN (20%), UA (73%), BGA (46%), and APA (26%) was noticed in T4 treatment without biochar (BS_0.5_ + CM_0.5_), as compared to control (T1). However, with biochar application, the highest increase in SMBC, SMBN, UA, BGA, and APA was 15%, 37%, 96%, 67%, and 45%, respectively, in T8 treatment representing consortium of biochar, biogas slurry, and compost (WSB_0.3_ + BS_0.3_ + CM_0.3_), as compared to control (T1).

### 2.6. Correspondence Analysis

**Figure 1:**
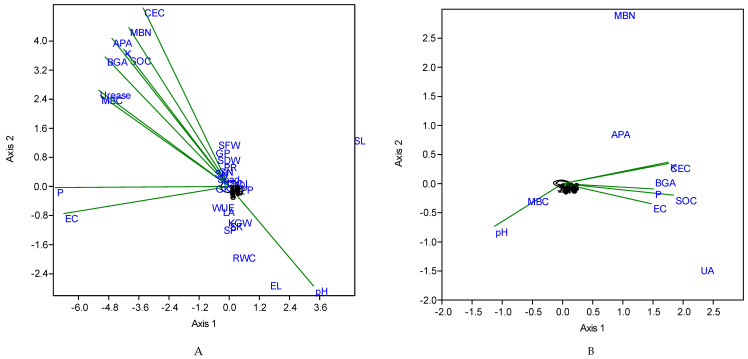
Correspondence analysis of soil physico-chemical and physiological parameters. In Figure 1A,B, the abbreviations are as CEC: cation exchange capacity, MBN: microbial biomass nitrogen, UA: urease activity, APA: alkaline phosphate activity, SOC: soil organic carbon, BGA: β-glucosidase activity, MBC: microbial biomass carbon, SFW: shoot fresh weight, SDW: shoot dry weight, PR: photosynthetic rate, GN: grain nitrogen, GP: grain phosphorus, GK: grain potassium, SN: straw nitrogen, SP: straw phosphorus, SK: straw potassium, KGW: 1000 grain weight, EC: electrical conductivity, P: phosphorus, SPAD: soil plant analysis development, WUE: water use efficiency, LA: leaf area, RWC: relative water content, EL: electrolyte leakage.

The improvement in soil properties after amendments corresponded with the soil enzyme activity. The results revealed that soil EC, SOC, P, K, and CEC best correlated with UA, BGA, and APA activity (axis-I (91%), axis-II (8%) (Figure 1A). While, pH, SOC, K, CEC, MBN, BGA, and APA best correlated with EL, SL, GP, and SFW of the plant (axis-I (76%), axis-II (19%) (Figure 1B).

## 3. Discussion

### 3.1. Effect of Organic Amendments on Soil Properties

Organic amendments help to enhance the SOC and productivity which ultimately improve plant growth [38]. Biochar application enhanced CEC, nutrients (K, P, Mg, Ca) uptake, improved soil quality and maize yield, as observed in the previous study by Sukartono et al. [39]. The combined use of biochar and manure compost application significantly reduced the soil pH when applied with a pyroligneous solution (BPC-PS) and 0.3-unit reduction in soil pH over control was observed after the addition of BPC-PS [35]. This slight manipulation in soil pH may be useful for nutrient uptake by enhancing nutrient mobilization from alkaline soil, as evidenced in our study. Sarwar et al. [40] also reported significant alteration in soil pH and SOM that enhanced the nutrient uptake from compost amended soil. However, contrasting results were noticed by Yuan et al. [41] that biochar addition significantly increased the soil pH. Similar findings were also noticed by Al-Wabel et al. [42] that biochar addition significantly enhanced the soil pH, CEC, and base saturation in highly weathered soil. While findings by Schulz et al. [43] indicated a non-significant alteration in soil pH when co-composted biochar was incorporated in the soil.

### 3.2. Effect of Organic Amendments on Nutritional Status of Maize

The sole and combined use of biochar and compost application enhanced the soil fertility due to nutrients composition of compost, and large surface area, high CEC, WHC, and pyrolysis temperature of biochar [44]. The sole application of organic amendments (biochar, compost, and biogas slurry) influenced the soil pH, EC, CEC, and provided essential nutrients (C, N, P, K, and S) to plant [45,46]. The improvement in soil fertility leads to enhanced plant growth via uptake of essential nutrients supplied by the organic amendments. Liu et al. [47] observed a synergistic relationship between biochar-compost on nutrient and WHC, and SOM in a field experiment. In our study, biochar improved plant growth by increasing the NUE, microbial growth, and water-holding capacity in the soil, as noticed in previous studies [48,49]. However, biochar feedstocks, pyrolysis temperature, and application rates played a significant role in the yield of the crop [50,51]. Biochar application affects various factors such as soil physiochemical properties, microbial growth, and nutrients biogeochemical cycles, especially C and N cycles in soil [52]. Biochar addition increased P availability [53] and K concentration resulting in higher plant growth [54]. Sarfraz et al. [55] observed increased P uptake in maize as a result of increasing biochar rate with and without N fertilizers. An increase in plant N content and maize growth by biochar application [56,57] may be due to the higher surface area of biochar that retains NH^4+^ in the soil and its high CEC [58]. It was observed by many studies that the combined application of biochar and other organic treatments increases nutrients availability and moisture retention that ultimately improves plant growth and soil quality [8,34,59].

### 3.3. Effect of Organic Amendments on Maize Growth Parameters

Soil management practices could improve soil health and enhance crop yield through different organic amendments. The results of our study demonstrated that the incorporation of biochar, compost, and biogas slurry has a beneficial effect on the agronomic parameters of maize. It is well documented that manure and compost additions enhanced the SOC content and other essential nutrients (P, K, Ca, Mg), as compared to un-amended soil [13]. Application of compost in soil improves soil structure resulting in reduced bulk density and increased soil aeration, water retention, and crop growth [14]. While biochar application slowly releases the available nutrients to plants in their whole growing period and reduced the leaching of nutrients (C, N, P, and K) after the addition of compost and slurry [60]. Butnan et al. [61] studied different rates of biochar (1, 2, and 4%) prepared at 350 °C in loam sand (pH = 5.5) and silty clay loam soil (pH = 6). They observed 115–600% increase in maize dry weight over control. Similarly, 62–113% increase in dry weight was noticed by Inal et al. [62] when biochar prepared at 300 °C was applied in clay loam soil (pH = 7.8) with different rates (0, 2.5, 5, 10, and 20 g kg^−1^). Ali et al. [63] study reported an increase of 23% in plant height and 17% in leaf area by biochar application. Many researchers noticed that biochar application in soil increased plant height, growth, and grain quality [64,65,66]. This increase may be due to improved nutrient and moisture content availability. The CCA results also confirmed that improved soil properties (pH, SOC, K, CEC, MBN, BGA, and APA) were the main drivers for enhancing plant growth and yield. Our results are similar to Uzoma et al. [67] that animal manure biochar addition to soil enhanced the WUE (91–139%) and maize yield (98–150%). In another study, compost and biochar application along with chemical fertilizer significantly increased chlorophyll content [68]. The soil amended with biochar, biogas slurry, and compost has high organic carbon, improved porosity, and enriched nutrients (P, K), as compared to control which could be the reason for improved crop growth and other physiological parameters.

### 3.4. Effect of Organic Amendments on SMB and Enzyme Activity 

Soil microbial biomass is a living component of the soil which is directly linked to the mineral nutrition and has been considered as a sensitive indicator for short-term nutritional and environmental changes in soil [69]. In addition, soil microbial activity plays significant role in soil health involving carbon and nutrient cycling and their sustainability [70,71]. The results of our study showed that organic inputs (biochar, compost, and biogas slurry) had a positive effect on the SMB and enzyme activity. Biochar application enhanced the SMB which might be due to several reasons such as nutrients availability to microbes [72], labile organic compounds [73], and stimulation of microbial activities on biochar particles through sorption of organic C [74]. Biochar surface has numerous pores that provide shelter to microbes against stress [75], which results in an increase in SMB. Similar findings were also observed in the previous studies which reported an increase in SMBC due to biochar application [76,77,78]. However, in contrast, no change in MBC under biochar application was observed by Bruun et al. [73]. Dempster et al. [79] noticed a reduction in MBC and MBN as a result of biochar addition in the soil.

Soil enzymes worked as soil fertility indicators and influenced the micro-environment of the soil [80,81]. Soil physicochemical properties [82], crop type [83], microbial population, and anthropogenic activities directly control the activities of enzymes in the soil [84] and biochar addition in soil enhance soil enzyme activities [85,86]. Our results showed that sole or combined application of biochar, compost, and biogas slurry increased the soil urease and β-glucosidase activity. This enhanced enzyme activity could be due to the increased substrate concentration or microbial use and other processes [87]. Various soil factors like texture, soil structure, moisture content, soil temperature, pH, and SOM influence enzyme activity in soil [88,89]. The correspondence analysis also revealed that improvement in soil properties such as EC, SOC, P, K, and CEC plays a dominant role in improving the soil enzyme activity. Pokharel et al. [90] also observed that different levels of biochar increased UA in the soil. In another study by Mehmood et al. [91], UA in the soil after 60 and 120 days of sugarcane residues biochar (10 t ha^−1^) application increased by 7.9 and 12.7% over control. Similarly, 2% biochar prepared at 500 °C increased β-glucosidase in sandy loam soil [80,92].

## 4. Materials and Methods 

### 4.1. Compost and Biogas Slurry Production

The compost was prepared from fruit and vegetable wastes in a locally fabricated composter unit. The fruits and vegetable wastes were collected from the market, and sun-dried to remove moisture and unwanted material especially plastic bags, stones, etc. After drying, organic wastes were grounded and put into a composter having 500 kg capacity. During composting, 60% moisture level was maintained for proper composting and rotated at 50 rpm for fifteen days. The finished product was odorless and dark brown in color. Biogas slurry (BS) is a secondary product produced in the process of biogas production. The slurry was collected from the biogas unit at the farmhouse of animal husbandry at the University of Agriculture, Faisalabad. The biochar, compost, and biogas slurry were characterized for the basic properties (Table 6).

### 4.2. Biochar Production and Characterization

For biochar production, wheat straw feedstock was obtained from the “Agronomy Research Field” and brought to the biochar furnace unit for biochar production. The feedstock was washed to remove dust, air-dried and crushed (<0.5 cm) until feedstock was filled in 2 liters (L) flask, made up of Pyrex glass which can bear a higher temperature up to 1000 °C. The bent glass rod was attached to a flask outlet with silicone grease for gases and water vapor removal. Pyrolysis process was carried out at 350 °C for biochar production with 10 °C min^−1^ increase in temperature, sustained for 30 min for proper pyrolysis [93] and left for cool down. Furnace lid was opened when the temperature was below 30 °C and biochar was stored after grinding (0.28 mm) in plastic bags for further processing. 

The standard procedures were followed for the characterization of biochar, compost, and biogas slurry (Table 1). Biochar, compost, and biogas slurry’s pH and electrical conductivity (EC) were measured by using 1:20 (solid: solution) with distilled water after shaking on a mechanical shaker (90 rpm). The modified NH_4_-acetate method was applied for CEC measurement [94]. The nutrient status of biochar, compost, and biogas slurry especially, nitrogen (N), phosphorous (P), and potassium (K) was determined by the protocol described by Wolf [95]. The wet digestion of biochar samples was carried out with sulphuric acid (H_2_SO_4_) and hydrogen peroxide (H_2_O_2_). After digestion, samples were preserved for analysis. Total nitrogen was determined with the Kjeldahl method [96]. The flame photometer (PFP7, Jenway, Essex, UK) was used for K determination and P was determined by spectrophotometer (UV-1201, Shimadzu, Tokyo, Japan), following vanadate-molybdate procedure [97].

### 4.3. Pot Experiment

A pot trial was conducted at the greenhouse of the “Institute of Soil and Environmental Science, University of Agriculture, Faisalabad, Pakistan” to observe the effect of different organic amendments on soil quality, growth and yield of maize plant. For this purpose, 30 kg air-dried sandy clay loam soil free of stones and stubbles (31.439082° N, 73.069365° E) was used in each pot. The recommended rate of N, P, and K (220:180:120 kg ha^−1^) was used by mixing urea, di-ammonium phosphate (DAP), and sulfate of potash (SOP) fertilizers, and were added to soil in each pot. Different levels of compost and biogas slurry with and without biochar were homogeneously blended in soil. Maize variety FH-1046 was used as a test crop and 5 seeds were sown on Feb-15, 2018 in each pot. The experiment was carried out with different treatment combinations as described below:

T1: Control (CK)
T2: Biogas slurry 1% (BS)
T3: Compost 1% (CM)
T4: Biogas slurry 0.5% + Compost 0.5% (BS_0.5_ + CM_0.5_)
T5: Wheat straw biochar 1% (WSB)
T6: Biochar 0.5% + Biogas slurry 0.5% (WSB_0.5_ + BS_0.5_)
T7: Biochar 0.5% + Compost 0.5% (WSB_0.5_ + CM_0.5_)
T8: Biochar 0.3% + Biogas slurry 0.3% + Compost 0.3% (WSB_0.3_ + BS_0.3_ + CM_0.3_)

### 4.4. Pre-Soil Analysis

The soil samples were homogenized for basic soil analysis, sealed in plastic bags to prevent contamination and brought to the laboratory for analysis. Soil pH was measured with a pH meter (JENCO Model- 671 p) from saturated soil paste. Soil extract was used for the determination of electrical conductivity (EC). EC meter was standardized with 0.01 N KCl solutions [98]. Soil texture determination was done by the hydrometer method described by Bouyoucos (1962) [99] and SOM was determined by following the standard method described by Moodie et al. (1959) [100]. Total N in the soil was measured using the Kjeldahl apparatus by taking 10 g soil in digestion flask and 40 mL of H_2_SO_4_ with digestion mixture (K_2_SO_4_. FeSO_4_. CuSO_4_). Soil available P was determined by using the extraction solution of 0.5 M NaHCO_3_ [101], at 880 nm wavelength by using a spectrophotometer (Milton Roy Company), while soil K was estimated after extraction with ammonium acetate (1 N) using a flame photometer (Jenway PFP-7) [98]. 

### 4.5. Plant Physiochemical Parameters

Photosynthetic rate (PR), water use efficiency (WUE), stomatal conductance (SC), transpiration rate (TR), and transpiration rate per unit of leaf area (EL) were measured by using a portable CIRAS-3 (PP Systems, Hitchin, UK). SPAD-502 (Minolta, Osaka, Japan) was used for chlorophyll content (CC) measurement. The relative water content (RWC) of the leaf was measured by using the following formula [102]:

RWC = Turgid Weight − Dry weight/Fresh weight − Dry weight(1)

### 4.6. Plant Nutritional Analysis

The nutrient analysis of maize was determined by taking sulfuric acid (H_2_SO_4_) and hydrogen peroxide (H_2_O_2_) as a digestion mixture [95]. In brief, digestion was carried out by taking 2 mL of conc. H_2_SO_4_ in digestion flask that contained 0.5 g plant sample, covered with aluminum sheet and left overnight. The next day, H_2_SO_4_ (1 mL) was added in digestion flasks and put on a hot plate in a fume hood for escape of vapors and fumes safely. Another 1 mL of H_2_O_2_ was added and the sample was heated until a clear solution was obtained. After digestion, the sample was filtered with filter paper and the final volume was made to 50 mL with distilled water and stored for further analysis.

### 4.7. Total Nitrogen Determination (%)

The Kjeldahl method was used for N determination. So, 5 mL of digested sample and 10 mL of 40% sodium hydroxide (NaOH) were poured into the ammonium distillation flask placed in the distillation setup. Then, 5 mL of boric acid (2%) and few drops of indicator (Bromocresol green+ methyl red) were taken in 100 mL conical flask and removed after receiving 35-40 mL of distillate and left for cooling for a few minutes. The sample was titrated against standard H_2_SO_4_ (0.01N) till the pink color endpoint was observed. To determine the percent nitrogen (% N), the following formula was used.

% N = (T*N*1.4) sample weight
Where, T = acid volume used, N = H_2_SO_4_ normality, and sample weight = 0.1 g.

### 4.8. Post-Harvest Soil Analysis

Soil samples of each treatment after harvesting were collected and stored for post-harvest soil analysis. Soil pH was determined by making a saturated paste and ECe _(1:20)_ was measured according to U.S. Salinity Laboratory Staff (1954) [98]. The Kjeldahl method was used to measure the total N and the Walkley-Black titration method (with K_2_Cr_2_O_7_-H_2_SO_4_ mixture for organic carbon oxidation) was used for soil organic carbon (SOC) determination [103].

### 4.9. Microbial Biomass Carbon (MBC) and Microbial Biomass Nitrogen (MBN)

The MBC and MBN in soil were estimated using the chloroform fumigation-extraction procedures (Brookes et al. 1985). For this, a moist soil sample (10 g) was taken in the crucible and fumigated with 30 mL of alcohol-free chloroform (CHCl_3_). While the other 10 g of sample was placed in desiccator without chloroform and placed at room temperature for 24 h [104]. The soil samples with 0.5 M K_2_SO_4_ (50 mL) were kept shaking on a horizontal shaker for 30 min with a speed of 200 rev min^−1^ that contained lysed microorganisms and were filtered further. The MBC was determined by using a standard curve at 600 nm wavelength on a spectrophotometer (UV-VIS/1201, Shimadzu), while MBN was measured with the Kjeldahl method. Both MBC and MBN were calculated by using the following equation [105]:

MBC (mg) = Ec/k
Ec = Extracted C produced as a result of fumigation
k = Fraction of biomass extracted as C under standard conditions and is 0.35 for C and 0.45 for N [106].

The soil urease activity (UA) was measured by taking 5 g of sample mixed with urea (2.5 mL) and KCl (50 mL) solution in a conical flask [107]. The sample was centrifuged at 180 rpm for 30 min and transferred into an incubator for 2 h at 37 °C. After incubation, 1:9 filtrate and ddH_2_O solution were prepared and 5 mL sodium salicylate/NaOH along with cyanide (2 mL) were added and cooled at room temperature. The samples were run at 690 nm wavelength for urease determination. The β-glucosidase (BGA) in soil was determined by following Eivazi and Tabatabai [108] method. Sample (1 g) was mixed with 0.25 mL toluene, 1 mL of ρ-nitrophenyl-β-glucosidase (PNG), and 4 mL of modified universal buffer (MUB) in 50 mL flask. After incubation at 37 °C for 1 h., 1 mL of CaCl_2_ (0.5 M), 4 mL buffer solution (hydroxyl methyl aminomethane solution) of pH 12, and sample were measured at wavelength of 400 nm [108]. Alkaline phosphatase activity (APA) was determined by taking 1 g of soil mixed with 1 mL *p*-nitrophenyl phosphate (*p*-NPP), toluene (0.25 mL), and 4 mL modified universal buffer (pH 11) and incubated. After incubation, 1 mL of CaCl_2_ (0.5 M) and 4 mL of NaOH (0.5 M) was added and filtered. The absorbance of sample was measured at 400 nm on a spectrophotometer (UV-VIS/1201, Shimadzu, CA, USA).

### 4.10. Statistical Analysis

The significant differences among treatments were analyzed using a two-way analysis of variance (ANOVA) by taking biochar as a factor using Statistix 8.1 software(Statistix, Tallahassee, FL, USA). The correspondence of soil variables (soil properties and enzymes) with plant variables (nutrient composition, physiology, and growth) was analyzed using canonical correspondence analysis (CCA) (Past 3.0).

## 5. Conclusions

Application of organic amendments to the soil is an environmental-friendly, easily available, and cost-effective method to increase nutrient use efficiency and to reduce chemical fertilizers up to 50%. The integrated use of biochar, compost, and biogas slurry improved soil carbon and nutrient status (N, P, and K), as well as restored soil fertility. Moreover, the combined use of all these organic amendments improved the plant length and weight (fresh and dry), RWC, WUE, and chlorophyll contents over their sole use. Here, biochar addition significantly reduced the nutrient losses of compost and biogas slurry. Thus, organic amendments of different composition (biochar, compost, and biogas slurry) can be potentially utilized in combination under the current scenario as a sustainable tool for improving the fertility of agricultural soils.

## Figures and Tables

**Table 1:** Effects of wheat straw biochar, compost, and biogas slurry on soil physico-chemical properties.

	Treatments	pH	EC (dS m^−1^)	CEC (cmol_c_ kg^−1^)	SOC (%)	P (mg kg^−1^)	K (mg kg^−1^)
Without biochar	T1 (CK)	7.92 ± 0.03 ^a^	1.14 ± 0.01 ^b^	6.13 ± 0.20 ^d^	0.49 ± 0.03 ^e^	4.44 ± 0.08 ^e^	84.48 ± 1.2 ^g^
	T2 (BS)	7.88 ± 0.02 ^ab^	1.30 ± 0.01 ^a^	6.40 ± 0.12 ^c^	0.60 ± 0.01 ^c^	6.82 ± 0.03 ^d^	98.18 ± 2.45 ^f^
	T3 (CM)	7.87 ± 0.02 ^ab^	1.30 ± 0.02 ^a^	6.23 ± 0.11 ^cd^	0.59 ± 0.01 ^cd^	6.95 ± 0.04 ^c^	100.17 ± 0.81 ^f^
	T4 (BS_0.5_ + CM_0.5_)	7.87 ± 0.02 ^ab^	1.29 ± 0.03 ^a^	6.22 ± 0.11 ^cd^	0.83 ± 0.02 ^b^	7.02 ± 0.01 ^bc^	112.97 ± 1.36 ^d^
With biochar	T5 (WSB)	7.85 ± 0.02 ^b^	1.28 ± 0.03 ^a^	7.26 ± 0.23 ^b^	0.54 ± 0.03 ^de^	7.10 ± 0.1 ^b^	103.53 ± 1.91 ^e^
	T6 (WSB_0.5_ + BS_0.5_)	7.87 ± 0.03 ^ab^	1.31 ± 0.01 ^a^	7.41 ± 0.14 ^b^	0.87 ± 0.05 ^ab^	7.05 ± 0.03 ^b^	130.13 ± 1.5 ^c^
	T7 (WSB_0.5_ + CM_0.5_)	7.88 ± 0.01 ^ab^	1.29 ± 0.01 ^a^	7.32 ± 0.13 ^b^	0.83 ± 0.03 ^b^	7.04 ± 0.01 ^b^	134.17 ± 1.53 ^b^
	T8 (WSB_0.3_ + BS_0.3_ + CM_0.3_)	7.77 ± 0.06 ^c^	1.31 ± 0.02 ^a^	8.03 ± 0.25 ^a^	0.90 ± 0.04 ^a^	7.42 ± 0.01 ^a^	153.29 ± 1.22 ^a^

Means sharing similar letter(s) in a column do not differed significantly at *p = 0.05*; Data is average of three replicates ± SE Different abbreviations are as follows: CK (control); BS (biogas slurry); CM (compost); WSB (wheat straw biochar); EC (electrical conductivity); CEC (cation exchange capacity); SOC (soil organic carbon).

**Table 2:** Effects of wheat straw biochar, compost, and biogas slurry on nutritional status of maize.

	Treatments	Straw N (g kg^−1^)	Straw P (mg kg^−1^)	Straw K (mg kg^−1^)	Grain N (g kg^−1^)	Grain P (mg kg^−1^)	Grain K (mg kg^−1^)
Without biochar	T1 (CK)	0.18 ± 0.03 ^d^	0.65 ± 1.95 ^d^	2.39 ± 0.44 ^c^	0.50 ± 0.05 ^d^	1.16 ± 3.47 ^e^	3.75 ± 0.07 ^f^
	T2 (BS)	0.23 ± 0.03 ^cd^	0.92 ± 2.76 ^bc^	2.98 ± 0.15 ^b^	0.65 ± 0.01 ^c^	1.43 ± 4.30 ^cd^	4.77 ± 0.02 ^e^
	T3 (CM)	0.24 ± 0.04 ^c^	0.89 ± 2.68 ^c^	3.03 ± 0.08 ^b^	0.63 ± 0.02 ^c^	1.40 ± 4.19 ^d^	5.60 ± 0.06 ^d^
	T4 (BS_0.5_ + CM_0.5_)	0.31 ± 0.01 ^ab^	0.92 ± 2.78 ^bc^	2.91 ± 0.24 ^b^	0.81 ± 0.02 ^b^	2.08 ± 6.23 ^b^	6.20 ± 0.04 ^b^
With biochar	T5 (WSB)	0.30 ± 0.03 ^ab^	0.93 ± 2.80 ^bc^	3.10 ± 0.11 ^b^	0.62 ± 0.01 ^c^	1.48 ± 4.44 ^c^	5.89 ± 0.01 ^c^
	T6 (WSB + BS_0.5)_	0.29 ± 0.06 ^bc^	0.94 ± 2.84 ^b^	3.10 ± 0.11 ^b^	0.82 ± 0.02 ^b^	2.04 ± 6.13 ^b^	6.24 ± 0.07 ^b^
	T7 (WSB + CM_0.5_)	0.29 ± 0.02 ^bc^	0.93 ± 2.79 ^b^	3.09 ± 0.20 ^b^	0.82 ± 0.01 ^b^	2.09 ± 11.67 ^b^	6.25 ± 0.03 ^b^
	T8 (WSB + BS + CM_0.3_)	0.35 ± 0.02 ^a^	1.02 ± 3.06 ^a^	3.55 ± 0.15 ^a^	0.93 ± 0.03 ^a^	2.23 ± 6.69 ^a^	7.18 ± 0.06 ^a^

Means sharing similar letter(s) in a column do not differed significantly at *p = 0.05*; Data is average of three replicates ± SE Different abbreviations are as follows: CK (control); BS (biogas slurry); CM (compost); WSB (wheat straw biochar); N (nitrogen); P (phosphorus); K (potassium).

**Table 3:** Effects of wheat straw biochar, compost, and biogas slurry on physiological parameters of maize.

	Treatments	RWC (%)	EL (%)	TR (mmol m^−2^ S^−1^)	CC (mg g^−1^)	SC (mmol m^−2^ S^−1^)	WUE (μmol m^−2^ s^−1^)
Without biochar	T1 (CK)	49.4 ± 0.70 ^e^	44.3 ± 1.01 ^a^	13.6 ± 1.21 ^d^	27.63 ± 1.34 ^e^	148.9 ± 2.03 ^d^	2.53 ± 0.15 ^c^
	T2 (BS)	65.2 ± 8.51 ^bc^	35.6 ± 2.81 ^c^	15.3 ± 0.72 ^cd^	37.53 ± 1.27 ^d^	163.8 ± 2.31 ^c^	4.00 ± 0.62 ^b^
	T3 (CM)	65.5 ± 1.21 ^bc^	39.3 ± 1.19 ^b^	16.2 ± 1.62 ^c^	35.00 ± 1.45 ^d^	166.0 ± 11.3 ^c^	3.87 ± 0.47 ^b^
	T4 (BS + CM_0.5_)	56.6 ± 4.02 ^de^	39.3 ± 0.62 ^b^	19.6 ± 1.14 ^b^	46.93 ± 2.54 ^b^	207.7 ± 2.97 ^b^	4.45 ± 0.49 ^ab^
With biochar	T5 (WSB	58.7 ± 1.71 ^cd^	39.8 ± 1.93 ^b^	16.3 ± 0.72 ^c^	41.87 ± 1.17 ^c^	165.4 ± 2.82 ^c^	3.87 ± 0.42 ^b^
	T6 (WSB + BS_0.5_	69.9 ± 1.72 ^ab^	38.5 ± 0.54 ^b^	19.3 ± 0.91 ^b^	42.00 ± 2.02 ^c^	201.5 ± 2.28 ^b^	4.77 ± 0.08 ^a^
	T7 (WSB + CM_0.5_	75.6 ± 4.31 ^a^	34.1 ± 1.62 ^c^	19.5 ± 1.84 ^b^	41.90 ± 1.87 ^c^	205.3 ± 7.25 ^b^	4.72 ± 0.14 ^a^
	T8 (WSB + BS + CM_0.3_)	60.5 ± 6.90 ^cd^	27.1 ± 1.43 ^d^	24.1 ± 0.45 ^a^	52.50 ± 3.30 ^a^	221.3 ± 1.91 ^a^	4.97 ± 0.02 ^a^

Means sharing similar letter(s) in a column do not differed significantly at *p = 0.05*; Data is average of three replicates ± SE Different abbreviations are as follows: CK (control); BS (biogas slurry); CM (compost); WSB (wheat straw biochar); RWC (relative water content); EL (electrolyte leakage); TR (transpiration rate); CC (chlorophyll content); SC (stomatal conductance); WUE (water use efficiency).

**Table 4:** Effects of wheat straw biochar, compost, and biogas slurry on maize growth attributes.

	Treatments	Plant Height (cm)	Plant Fresh Weight/Pot (g)	Plant Dry Weight/Pot (g)	Leaf Area (cm^2^ g^−1^)	Cob Length (cm)	Total Grain Yield/Pot (g)	1000 Grain Weight (g)
Without biochar	T1 (CK)	118 ± 4 ^e^	202 ± 7.51 ^f^	64 ± 2.74 ^e^	64 ± 7.56 ^f^	4.13 ± 0.12 ^c^	132 ± 0.95 ^e^	295 ± 13.01 ^d^
	T2 (BS)	144 ± 4 ^d^	236 ± 17.80 ^e^	81 ± 2.65 ^d^	84 ± 8.21 ^e^	5.23 ± 0.38 ^b^	147 ± 4.48 ^d^	370 ± 15.52 ^c^
	T3 (CM)	146 ± 4 ^d^	230 ± 13.83 ^e^	79 ± 3.66 ^d^	82 ± 4.97 ^e^	5.07 ± 0.31 ^b^	160 ± 5.57 ^c^	376 ± 17.03 ^c^
	T4 (BS_0.5_ + CM_0.5_)	170 ± 8 ^ab^	339 ± 9.25 ^b^	108 ± 6.67 ^b^	87 ± 8.06 ^d^	5.37 ± 0.23 ^b^	195 ± 2.33 ^a^	408 ± 12.12 ^ab^
With biochar	T5 (WSB)	151 ± 4 ^cd^	287 ± 10.32 ^d^	84 ± 4.80 ^d^	90 ± 8.10 ^c^	5.33 ± 0.42 ^b^	164 ± 6.70 ^bc^	384 ± 12.74 ^bc^
	T6 (WSB_0.5_ + BS_0.5_)	170 ± 6 ^ab^	308 ± 11.40 ^cd^	100 ± 1.57 ^c^	93 ± 3.18 ^b^	5.27 ± 0.25 ^b^	170 ± 6.09 ^b^	394 ± 14.57 ^bc^
	T7 (WSB_0.5_ + CM_0.5_)	164 ± 8 ^bc^	315 ± 6.55 ^bc^	105 ± 4.01 ^bc^	89 ± 3.56 ^d^	5.23 ± 0.25 ^b^	166 ± 5.70 ^bc^	391 ± 15.14 ^bc^
	T8 (WSB_0.3_ + BS_0.3_ + CM_0.3_)	182 ± 5 ^a^	386 ± 8.22 ^a^	123 ± 3.05 ^a^	98 ± 8.69 ^a^	7.27 ± 1.08 ^a^	200 ± 3.10 ^a^	423 ± 14.50 ^a^

Means sharing similar letter(s) in a column do not differed significantly at *p = 0.05*; Data is average of three replicates ± SE CK (control); BS (biogas slurry); CM (compost); WSB (wheat straw biochar).

**Table 5:** Effects of wheat straw biochar, compost, and biogas slurry on soil quality parameters.

	Treatments	SMBC (mg kg^−1^ Soil)	SMBN (mg kg^−^^1^ Soil)	Urease (µg g^−1^ dwt h^−1^)	B-Glucosidase (µg g^−1^ dwt h^−1^)	Alkaline Phosphates (µg g^−1^ dwt h^−1^)
Without biochar	T1 (CK)	217 ± 2.63 ^c^	21.4 ± 0.90 ^d^	23.1 ± 0.26 ^h^	11.4 ± 0.04 ^f^	18.0 ± 0.30 ^f^
	T2 (BS)	232 ± 2.31 ^b^	23.8 ± 1.17 ^c^	35.0 ± 0.10 ^e^	14.2 ± 0.01 ^d^	20.1 ± 0.25 ^e^
	T3 (CM)	229 ± 2.45 ^b^	23.9 ± 0.40 ^c^	33.2 ± 0.43 ^f^	14.1 ± 0.04 ^d^	19.8 ± 0.18 ^e^
	T4 (BS_0.5_ + CM_0.5_)	233 ± 2.79 ^b^	26.7 ± 0.66 ^b^	40.0 ± 0.10 ^b^	16.6 ± 0.05 ^b^	22.6 ± 0.17 ^c^
With biochar	T5 (WSB)	232 ± 3.16 ^b^	26.3 ± 1.06 ^b^	28.2 ± 0.26 ^g^	13.5 ± 0.02 ^e^	21.2 ± 1.03 ^d^
	T6 (WSB_0.5_ + BS_0.5_)	233 ± 3.28 ^b^	25.7 ± 0.61 ^b^	38.2 ± 0.20 ^d^	15.6 ± 0.03 ^c^	22.9 ± 0.13 ^bc^
	T7 (WSB_0.5_ + CM_0.5_)	234 ± 3.70 ^b^	26.3 ± 0.85 ^b^	39.1 ± 0.59 ^c^	16.7 ± 0.02 ^b^	23.5 ± 0.46 ^b^
	T8 (WSB_0.3_ + BS_0.3_ + CM_0.3_)	249 ± 3.99 ^a^	33.7 ± 1.57 ^a^	45.3 ± 0.20 ^a^	18.9 ± 0.38 ^a^	26.1 ± 0.36 ^a^

Means sharing similar letter(s) in a column do not differed significantly at *p = 0.05*; Data is average of three replicates ± SE Different abbreviations are as follows: CK (control); BS (biogas slurry); CM (compost); WSB (wheat straw biochar); SMBC (soil microbial biomass carbon); SMBN (soil microbial biomass nitrogen).

**Table 6:** Characterization of soil, wheat straw biochar, compost, and biogas slurry.

Parameters	Soil	Wheat Straw Biochar	Compost	Biogas Slurry
pH	7.95 ± 0.03	7.03 ± 0.16	7.53 ± 0.05	7.43 ± 0.02
Electrical conductivity (dS m^−1^)	1.15 ± 0.01	0.89 ± 0.02	3.05 ± 0.05	2.93 ± 0.02
Cation exchange capacity (Cmol_c_ kg^−1^)	5.79 ± 0.62	45.53 ± 0.84	--	--
Total nitrogen (%)	0.04 ± 0.003	1.38 ± 0.08	1.52 ± 0.03	1.98 ± 0.01
Available P (%)	4.01 ± 0.60	0.45 ± 0.02	0.31 ± 0.02	1.64 ± 0.01
Extractable K (%)	85.4 ± 2.34	1.06 ± 0.04	1.61 ± 0.01	1.43 ± 0.01
Ash content	--	25 ± 1.02	--	--
C/N ratio	--	38 ± 1.82	13±0.82	31 ± 1.02
Sand	49.80 ± 0.43	--	--	
Silt	27.57 ± 0.51	--	--	--
Clay	21.20 ± 0.88	--	--	--
Textural class	Sandy clay loam	--	--	--

Data is average of three replicates ± SE.
